# Arabinogalactan-Proteins as Boron-Acting Enzymes, Cross-Linking the Rhamnogalacturonan-II Domains of Pectin

**DOI:** 10.3390/plants12233921

**Published:** 2023-11-21

**Authors:** Rifat Ara Begum, Stephen C. Fry

**Affiliations:** 1 Department of Biochemistry and Molecular Biology, University of Dhaka, Dhaka 1000, Bangladesh; rifatab18@du.ac.bd; 2 The Edinburgh Cell Wall Group, Institute of Molecular Plant Sciences, The University of Edinburgh, Daniel Rutherford Building, The King’s Buildings, Max Born Crescent, Edinburgh EH9 3BF, UK

**Keywords:** acid-growth, arabinogalactan-proteins, borate diesterase, boron, Ca^2+^, chaperones, Pb^2+^, pectin, rhamnogalacturonan-II, trimers of RG-II

## Abstract

Most pectic rhamnogalacturonan-II (RG-II) domains in plant cell walls are borate-bridged dimers. However, the sub-cellular locations, pH dependence, reversibility and biocatalyst involvement in borate bridging remain uncertain. Experiments discussed here explored these questions, utilising suspension-cultured plant cells. In-vivo pulse radiolabelling showed that most RG-II domains dimerise extremely quickly (<4 min after biosynthesis, thus while still intraprotoplasmic). This tallies with the finding that boron withdrawal causes cell wall weakening within 10–20 min, and supports a previously proposed biological role for boron/RG-II complexes specifically at the wall/membrane interface. We also discuss RG-II monomer ↔ dimer interconversion as monitored in vitro using gel electrophoresis and a novel thin-layer chromatography method to resolve monomers and dimers. Physiologically relevant acidity did not monomerise dimers, thus boron bridge breaking cannot be a wall-loosening mechanism in ‘acid growth’; nevertheless, recently discovered RG-II trimers and tetramers are unstable and may thus underpin reversible wall loosening. Dimerising monomers in vitro by B(OH)_3_ required the simultaneous presence of RG-II-binding ‘chaperones’: co-ordinately binding metals and/or ionically binding cationic peptides. Natural chaperones of the latter type include highly basic arabinogalactan protein fragments, e.g., KHKRKHKHKRHHH, which catalyse a reaction [2 RG-II + B(OH)_3_ → RG-II–B–RG-II], suggesting that plants can ‘enzymically’ metabolise boron.

## 1. Introduction

### 1.1. Boron in Biology

Vascular plants are unusual among living organisms in exhibiting a well-defined and absolute requirement for the element boron (B). In the absence of a continual supply of B, plants are not able to continue growing and will die [1]. Most algae, animals and microbes on the other hand are not similarly dependent on B and any effects of traces of B appear to be beneficial rather than essential [2]. There is no clear evidence that bryophytes (liverworts etc.) require B [3], though traces of pectic-associated B are detectable in bryophyte cell walls [4].

Cell suspension cultures of ‘Paul’s Scarlet’ rose (a *Rosa* hybrid) [5], like those of *Chenopodium album* [6]*,* are unusual in being able to grow indefinitely in the absence of (detectable) B. This ability makes these cultures a valuable experimental tool for studying B biochemistry in vivo. However, no intact vascular plants have ever been demonstrated to be able to complete their life cycle in the absence of B.

The one conclusively demonstrated biochemical role of B in plants is to serve as a covalent cross-link between the rhamnogalacturonan-II (RG-II) domains of pectin in the primary cell wall. Beyond that, there is evidence that B is beneficial for plasma membrane function, enabling membrane–wall attachment [7,8,9,10,11] and enhancing nutrient uptake [12,13,14], but no clear-cut biochemical mechanism for this has been proven.

The sudden withdrawal of B from the medium of hydroponic roots or cell suspension cultures (by rinsing in zero-B media and/or application of the B ‘chelator’ azomethine H) leads to rapid changes in cell wall architecture, as judged for example by wall elasticity. Such effects can be seen within 10 to 20 min [15,16,17]. There are later knock-on effects on membrane properties, superoxide production, phenolic metabolism and cell death, but the first effect seems to be purely wall-related. Given that RG-II–B–RG-II bridges appear to be permanent in vivo, this observation indicates that the most recently produced RG-II domains are particularly crucial for cell wall strength. Amazingly, this implies that the most recent 10–20 min’-worth of RG-II domains (in the order of 1% of the total RG-II, assuming a cell doubling time of ~24 h) hold the key to overall wall elasticity, with the other 99% being less relevant or even possibly irrelevant. These considerations focus attention on an overriding biological role of B bridges in the pectin molecules located at the membrane–wall interface (i.e., those most recently synthesised and secreted). In other words, the B cross-linking of RG-II is a process that must be continually maintained on an essentially minute-by-minute basis in the living plant cell. This is a concept underlining the conclusion of Warington that “in water culture a continual supply of boric acid appears to be essential to healthy growth” and “a supply is required throughout the life of the plant” [1].

This article explores the mechanism by which B cross-links RG-II in vivo, whether it is reversible, and why this cross-linking is so important to the life of the plant.

### 1.2. RG-II: The Biosphere’s Most Sophisticated Polysaccharide Domain

**Figure 1:**
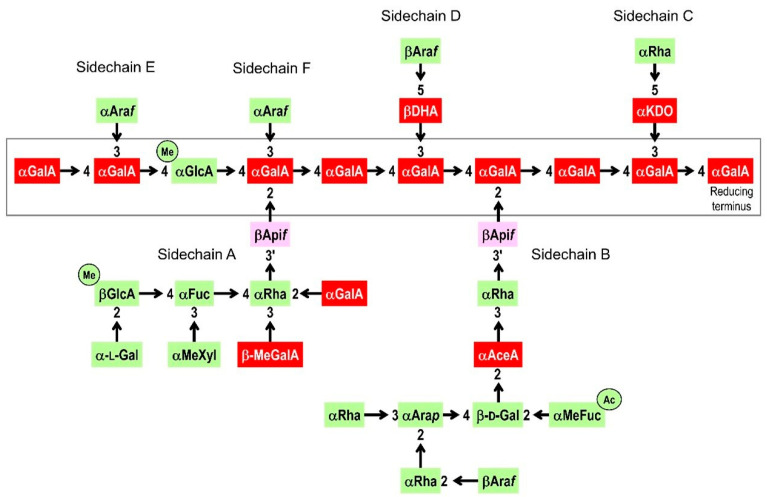
Primary structure of rhamnogalacturonan-II (RG-II) (adapted from references [18,19,20]). Abbreviations: Ac (in a circle), *O*-acetyl ester group; AceA, l-aceric acid; Api, d-apiose; Ara*f*, l-arabinofuranose; Ara*p*, l-arabinopyranose; DHA, 3-deoxy-d-*lyxo*-heptulosaric acid; Gal, galactose (d- or l-, as stated on the diagram); GalA, d-galacturonic acid; GlcA, d-glucuronic acid; KDO, 3-deoxy-d-*manno*-octulosonic acid; Me (in a circle), methyl ester group (attached to position 6, so that the uronic acid residue is not anionic); MeFuc, 2-*O*-methyl-l-fucose; MeGalA, d-galacturonic acid with methyl ether group(s) possibly on the 3- and/or 4-positions [20]; MeXyl, 2-*O*-methyl-d-xylose; Rha, l-rhamnose. All sugar residues are in the pyranose ring form unless indicated ‘*f*’ for furanose. The numerals (2, 3, 3′, 4, 5) indicate the position to which the neighbouring sugar residue makes a glycosidic bond; this bond is from the anomeric carbon (i.e., from C-1 of most sugars but from C-2 of the ketoses (DHA and KDO)). Arrows represent glycosidic bonds. Residues shaded red carry a negative charge at physiological pH (two negative charges in the case of DHA); those shaded green or purple are non-ionic; those shaded purple are the sites of boron binding. The grey rectangle represents the homogalacturonan-like backbone of RG-II.

RG-II is one of the three or four covalently linked domains of pectin in the primary cell walls of land plants, the others being RG-I, homogalacturonan (HG), sometimes xylogalacturonan and (in duckweeds and relatives) apiogalacturonan. RG-II is by far the most complex pectic domain, and possibly the most complex of all known polysaccharides in the biosphere. Its structure is highly (though not absolutely) conserved throughout the Embryophyta (land plants) [4]. RG-II, with a molecular weight of ~5000 and comprising ~30–33 sugar residues, has a backbone of about eight to ten α-(1 → 4)-d-galacturonic acid (GalA) residues (thus effectively being a short HG chain), one of which may carry a methyl ester group. The backbone is strongly anionic (negatively charged) at physiological pH. The primary structure of RG-II, i.e., its sequence of monosaccharide residues, has been defined in detail (Figure 1). To the backbone are attached five or six side chains, four of which are themselves also anionic. Thus, the whole RG-II molecule is highly anionic, carrying about 15 negative charges at pH 7.

**Figure 2:**
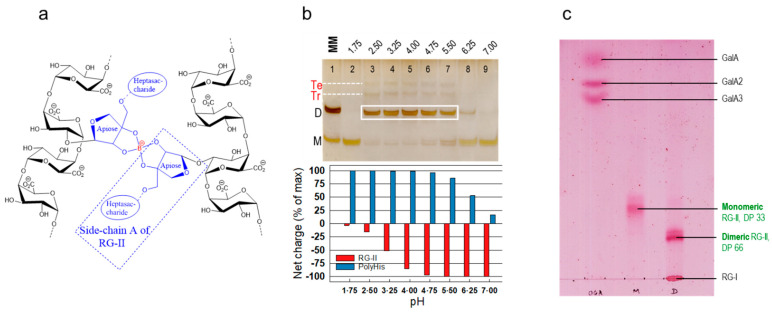
Boron bridging of RG-II. (**a**) Schematic representation of the B-bridging of two RG-II molecules [24]. The two sugar chains shown in black are portions of the backbones (effectively, short homogalacturonans) of these two RG-IIs. A side chain ‘A’ is attached via its apiose residue to each backbone, and a tetrahedral B atom (shown in red) can bond to two apiose residues, forming borate diesters and thereby dimerising the RG-II. (**b**) Effect of pH on peptide-mediated RG-II dimerisation, as revealed by polyacrylamide gel electrophoresis [23]. Eight reaction mixtures each contained 16 µM RG-II monomer, 1.2 mM boric acid, a 50 mM buffer, and a low concentration of a chaperone (0.55 µM polyhistidine). The pH values of the reaction mixtures are shown above the gel. After incubation at 20 °C for 16 h, the products were analysed by PAGE and stained with silver. The main dimer bands are highlighted by the white box. The putative RG-II trimer (Tr) and tetramer (Te) are also labelled. The marker mixture (MM, lane 1) contained 0.8 µg each of RG-II monomer (M) and dimer (D). The histogram shows the calculated effect of pH on the net charge of the polyhistidine (positive charges, blue bars) and RG-II (negative charges, red bars). Adapted with permission from Ref. [23], ©2023, Wiley Blackwell. (**c**) Thin-layer chromatography (TLC) as an alternative to PAGE for resolving monomers from dimers (unpublished). The samples loaded were (left to right) (i) oligogalacturonides, (ii) RG-II monomer, (iii) a mixture of RG-II dimer plus RG-I. The plastic-backed TLC plate was Merck silica gel. The plate was pre-washed sequentially by gentle rocking in acetone/acetic acid/water (1:1:1, 30 min, to remove thymol-stainable material from the plate), acetone (×2, each 15 min, to remove the acetic acid), 2% pyridine in acetone (15 min, to neutralise residual traces of acidity), and acetone (15 min, to remove traces of pyridine and expedite drying), dried, then baked in an oven for 30 min at 120 °C. The aqueous samples were loaded, then thoroughly dried in a stream of air (room temperature), and the chromatogram was developed (same day) in freshly prepared butan-1-ol/acetic acid/water (13:10:17 by vol.). The separated bands were stained in thymol/H_2_SO_4_ [21]. Previously unpublished work. Abbreviation: DP, degree of polymerisation.

One of the side chains (an octasaccharide known as chain A) is of particular interest as it is the best-established site to which boron may be attached. Specifically, boric acid can esterify to the –OH groups on carbons 2 and 3 of the apiose residue of side chain A. Moreover, the B atom can be tetrahedral, simultaneously forming four ester bonds: to the –OH groups of carbons 2 and 3 of the apiose of side chain A of each of two RG-II molecules (Figure 2a). In this way, a B atom can covalently cross-link two RG-II molecules. The product is an RG-II dimer, molecular weight ~10 kDa, which is highly stable at pH values above about 1.5 (at room temperature). Monomeric and dimeric RG-II can be separated and detected by polyacrylamide gel electrophoresis followed by silver staining (Figure 2b). We have also recently devised a TLC system for the same purpose (Figure 2c), which has the advantage of being able to run numerous samples simultaneously and with more quantitative staining (with thymol/H_2_SO_4_; [21]). With silver-staining on PAGE, in contrast, the dimer is detected with much greater sensitivity than an equal mass of monomer [5,22]. As will be discussed later, recent evidence suggests that B may also form weak bridges via the side chain B of RG-II [23]; their weakness is of particular interest as this type of B bridging would be reversible in vivo, unlike the stable bridging via side chain A.

For experimental purposes, the B-bridged 10-kDa dimer of RG-II can be readily hydrolysed to the 5-kDa monomer (plus boric acid) by incubation at pH 1 (0.1 M HCl) at room temperature

RG-II–B–RG-II + 2 H_2_O → 2 RG-II + boric acid

and after neutralisation the boric acid can be removed from the monomeric RG-II with 3.5-kDa cut-off dialysis tubing or by column chromatography on Bio-Gel P-2. After monomerisation, RG-II is kept in plastic vessels because glass may release traces of B compounds into aqueous media and slowly re-dimerise the RG-II.

Quantitatively, RG-II is a relatively minor component of the primary cell wall in land plants, and B-bridged RG-II constitutes 0.5–3.6% of the dry weight of dicot cell walls. The corresponding figures are 0.3–0.5% for grasses and cereals, 0.2–2.0% for ferns and lycopodiophytes, and only ~0.01% for bryophytes [4]. Aquatic monocots, especially members of the subfamily Lemnoideae (duckweeds, e.g., *Lemna minor* and *Spirodela polyrhiza*) have been reported to contain B-bridged RG-II [25]. RG-II has not been clearly demonstrated to occur in algae, even in the late-diverging charophytes that are the closest relatives of land plants, although the possibility of RG-II in algae is still under investigation.

Free RG-II does not occur in plant cell walls (either with or without B cross-linking). The evidence for this is that even though RG-II is highly water-soluble, aqueous buffers fail to extract any 5- or 10-kDa RG-II from the cell wall. It might be argued that any free RG-II would have been lost during cell wall isolation; however, free RG-II is also absent in the spent media of cell-suspension cultures [23], where any naturally free RG-II would have accumulated. The only known way of obtaining free RG-II is artificially—to digest the cell wall’s pectin enzymically. This appears to occur unintentionally during the production of wine, which has been reported to contain free dimerised RG-II [26,27,28,29,30]. With more control, it can be achieved with endo-polygalacturonase (EPG; pectinase), which hydrolyses the HG domain (after de-esterification of the latter in mild alkali) to highly soluble mono-, di- and trisaccharides, and releases RG-II (monomer and dimer) plus RG-I. Alternatively, pectin can be digested with Driselase, which hydrolyses HG and RG-I to monosaccharides, leaving RG-II intact but soluble. These observations suggest that HG, RG-II, and possibly also RG-I, are part of the same large pectin molecule, with a structure such as

…HG–RG-II–HG–RG-I–HG–RG-I–HG–…

which is insolubilised within the cell wall by various cross-links, e.g., Ca^2+^ bridges [31,32,33], covalent bonds to xyloglucan [34], and potentially pectin–glycoprotein complexes [35,36,37,38], phenolic cross-links [39,40,41,42] and physical entanglement. There have been no reports of an RG-II–RG-I complex among the EPG digestion products, suggesting that sequences such as …RGI–RG-II…, uninterrupted by HG, do not occur.

Usually, in the walls of healthy plant cells, the vast majority of the RG-II domains are in the B-bridged dimeric form, and are released as such by EPG. Exceptions are in B-starved cells and in mutant plants that have a mutated RG-II side-chain A (e.g., possessing l-Gal in place of the l-Fuc residue) [19,43,44], in which the RG-II monomer may predominate.

## 2. How Do Plants Dimerise RG-II?

### 2.1. Cations Can ‘Chaperone’ RG-II

#### 2.1.1. Background

How plants put the B in RG-II is a botanical enigma. Usually, adequate B is available to plants in the form of boric acid [designated H_3_BO_3_ or B(OH)_3_], dissolved in soil water. The *p*K_a_ of boric acid is about 9.2, so at typical soil pH values, which are <7, it is present in the uncharged form (thus not ‘borate’). Certain alcohols (R–OH), especially carbohydrates, are also present in soils [45,46,47,48,49,50,51], and it may be that these substances reversibly form negatively charged borate esters with boric acid, e.g.,

B(OH)_3_ + R–OH ↔ (OH)_3_–B^−^–O–R + H^+^

However, free boric acid is probably the form taken up by roots [45].

If monomeric RG-II is simply mixed with boric acid in aqueous solution in vitro, very little B bridging occurs and the RG-II remains monomeric. Evidently, mechanism(s) exist to facilitate B bridging in vivo, and this Section explores what such mechanism(s) might entail.

Potentially contributing factors are that the dimerisation reaction might

- be enabled by a catalyst, even an enzyme;
- depend on a ‘B donor’ substrate molecule;
- require the RG-II to be linked to a biological substance such as Ca^2+^ or RG-I.

The reaction under consideration can be simply formulated as

**Figure 3:**
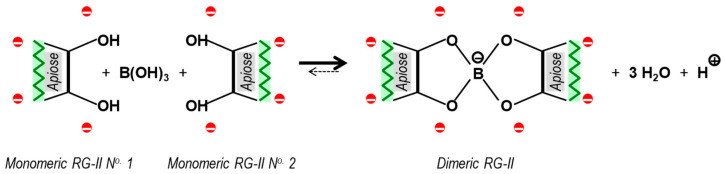
The essential reaction involved in boron bridging of RG-II [22]. Part of the neutral apiose residue of side chain A (in each of two monomeric RG-II molecules) is shown. The charges of its four near-neighbouring anionic sugar residues are rendered as red circles (two α-GalA residues in the RG-II backbone plus an α-GalA and a β-GalA residue attached to the rhamnose adjacent to the apiose). Monomeric RG-II has a total of about 11 additional anionic sugar residues, giving it a net charge of about −14 at the pH (4.8) used in our experiments. It is evident that the close approach of two RG-II monomers for B bridging requires the overcoming of considerable electrostatic repulsion; in addition, dimerisation introduces an additional negative charge on the previously neutral B atom (Adapted with permission from Ref. [22], ©2022, Portland Press).

2 RG-II(OH)_2_ + B(OH)_3_ → RG-II(O)_2_>B^−^<(O)_2_RG-II + 3 H_2_O + H^+^

(Figure 3) where RG-II(OH)_2_ represents an RG-II molecule with two –OH groups of its side chain A apiose residue shown, and (O)_2_>B^−^ represents two oxygens attached to one negatively charged B atom. This reaction is expected to be slow because the two interacting RG-II molecules are strongly negatively charged at physiological pH and thus liable to repel one another, hindering dimerisation. The negative charges on RG-II can be essentially abolished if the pH of the reaction mixture is reduced to about 2 (a pH at which B bridges are stable) or below. However, even at pH 2, B(OH)_3_ alone does not dimerise RG-II in vitro [23]. Therefore the reluctance of RG-II to dimerise with B(OH)_3_ in vitro cannot be attributed solely to electrostatic repulsion.

**Figure 4:**
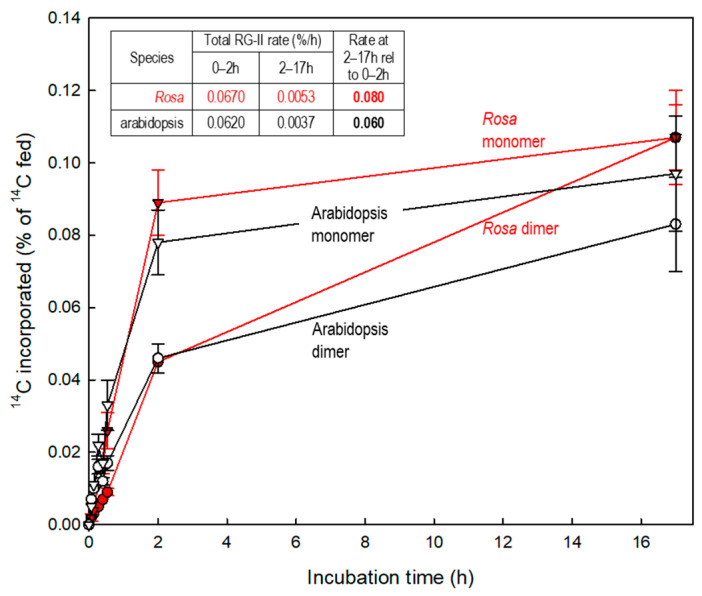
^14^C-Labelling of monomeric and dimeric RG-II domains in suspension-cultured rose and arabidopsis cells [52]. A pulse of [^14^C]glucose, supplied at time 0, was consumed by the cells within ~1–2 h, during which time the radioactivity was incorporated into cell wall polysaccharides including RG-II. At intervals, the monomeric and dimeric RG-II domains were assayed for ^14^C. Incorporation of ^14^C into the monomer (triangles) occurred during the first 2 h, and then almost stopped. Dimer radiolabelling (circles) mirrored this closely during the first 2 h, indicating that any given newly synthesised RG-II domain was highly likely to become dimerised almost immediately. But between 2 and 17 h, during which interval the vast majority of the radiolabelled cohort of molecules would have been in the cell wall, some additional dimer labelling continued, indicating slight RG-II dimerisation within the cell wall. Adapted with permission from Ref. [52], ©2022, Oxford University Press).

In living plant cells, RG-II domains are dimerised very soon after their synthesis [5]. Various approaches, including the use of in-vivo radiolabelling, show that the majority of the B bridging process occurs either intraprotoplasmically or immediately after secretion into the apoplast [5], mainly the former [52]. An additional small proportion of the eventual total dimerisation occurs in the cell wall, some hours or days after a given RG-II domain has been secreted (Figure 4).

What factors are present in vivo that enable B bridging to occur so rapidly when it is extremely slow in vitro? The most plausible way to overcome the repulsion between two anionic RG-II molecules is to ‘chaperone’ them to cations to form an approximately neutral complex enabling the two RG-II molecules to approach each other. Two options for doing this are described below, the second of which leads to the revolutionary idea of the first reported B-acting enzyme. [We would not choose to use the term ‘enzyme’ for the first option, which features metal cations rather than proteins.]

#### 2.1.2. Co-Ordinate Bonds via Divalent Metal Ions

**Figure 5:**
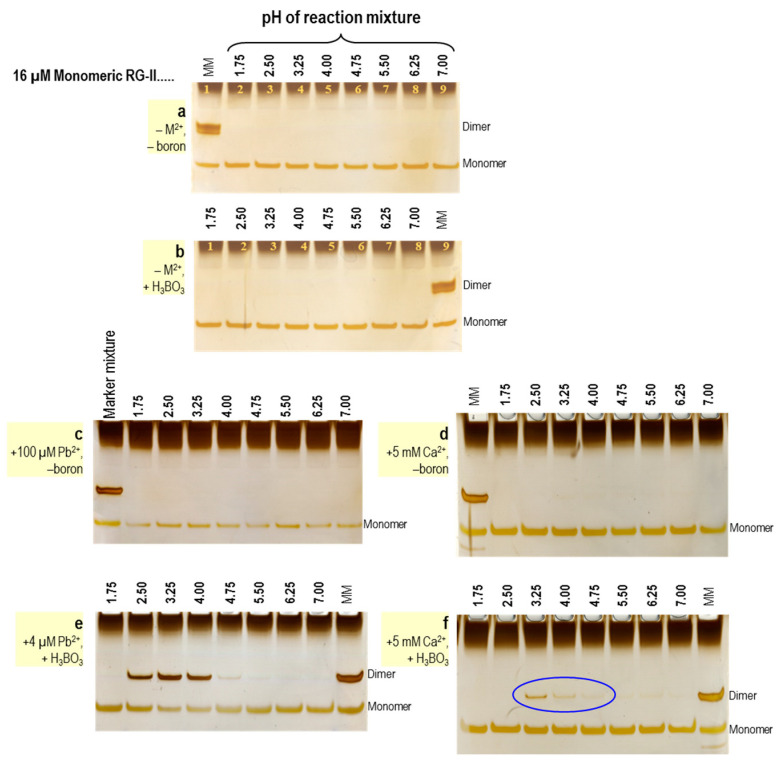
Inorganic cations as RG-II chaperones [23]. Each reaction mixture initially contained 16 µM pure monomeric RG-II and a 50 mM buffer suitable for the pH indicated above the gel lanes. (**a**) With no additional components; (**b**) with added 1.2 mM boric acid but no divalent cations; (**c**) with 100 µM Pb^2+^ but no B(OH)_3_; (**d**) with 5000 µM Ca^2+^ but no B(OH)_3_; (**e**) with 4 µM Pb^2+^ plus 1.2 mM boric acid; (**f**) with 2000 µM Ca^2+^ plus 1.2 mM boric acid. After 16 h incubation, the products were analysed by PAGE, resolving monomeric and dimeric RG-II. The blue ellipse in (**f**) highlights the (weak) ability of calcium to produce the dimer. Note that Pb^2+^ is >1000× more effective than Ca^2+^. MM, marker mixture of monomeric and dimeric RG-II for reference. Adapted with permission from Ref. [23], ©2023, Wiley Blackwell.

Although the above reaction (Figure 3) occurs only very slowly in vitro at all pH values tested (~1.75–7.0) [23], it can be greatly expedited by the addition of traces of certain ‘non-biological’ divalent metal cations, especially those with an ionic radius of >0.11 nm, e.g., Pb^2+^, Sr^2+^ and Ba^2+^ [7,31,32,53,54]. Pb^2+^ was highly effective at pH ~2–4, but not at higher pH values (Figure 5e) [23]. B bridging of RG-II can also be promoted by unphysiologically high concentrations of certain ‘biological’ metal ions, e.g., 50 mM Ca^2+^ [53], and slightly by 5 mM Ca^2+^ (but only within the narrow pH range of 3–4 [23]) (Figure 5f). A demonstration of the huge difference in efficiency of ‘non-biological’ Pb^2+^ and ‘biological’ Ca^2+^ for RG-II dimerisation is presented in Figure 5.

In principle, positively charged ions such as Pb^2+^ could bind ionically to RG-II, cancelling its negative charge and thereby facilitating dimerisation. However, two pieces of evidence argue against this explanation: (i) as mentioned above, merely neutralising the negative charges of RG-II does not cause B bridging, and (ii) Pb^2+^ is effective even at very low Pb:RG-II ratios (e.g., 0.25:1, mol/mol) which are far too low to cancel the net negative charge (−15) of a monomeric RG-II molecule. Thus, metal cations must promote RG-II dimerisation by a mechanism other than simply cancelling the electrostatic repulsion.

Such a mechanism is likely to be an ability of one Pb^2+^ ion to transiently link to two RG-II molecules, bringing them close enough together for a B bridge to form. This linkage is likely to be via co-ordinate bonding to form an RG-II–Pb^2+^–RG-II complex. The transient nature of this linkage is indicated by the fact that, when a mixture of monomeric RG-II and Pb^2+^ (in the absence of B) is subjected to gel electrophoresis, it co-migrates with pure 5-kDa monomer rather than with the 10-kDa dimer [23]. Although of theoretical interest, the effects of Pb^2+^ cannot be of direct biological relevance, except perhaps in understanding heavy-metal pollution, since Pb is not an essential element for plants.

An in-vivo alternative to Pb^2+^ could be Ca^2+^. This does not dimerise RG-II efficiently even at the high concentration of 5 mM, although it remains possible that it might do so in the presence of other biological agents (such as attached homogalacturonan) in vivo.

#### 2.1.3. Ionic Bonding via Organic Cations

Of more direct biological relevance as RG-II ‘chaperones’ could be organic cations, e.g., positively charged (glyco)proteins, which would interact ionically with negatively charged RG-II and potentially bring two RG-II molecules close enough for B bridges to be formed. As with the addition of metal ions and with the cancellation of the negative charges by buffering at low pH, the organic cations would not be expected to work by simply neutralising the net negative charge of the RG-II. More probably, they serve as a positively charged site to which two polyanionic RG-II molecules can simultaneously bond ionically, thus being seated close together.

**Figure 6:**
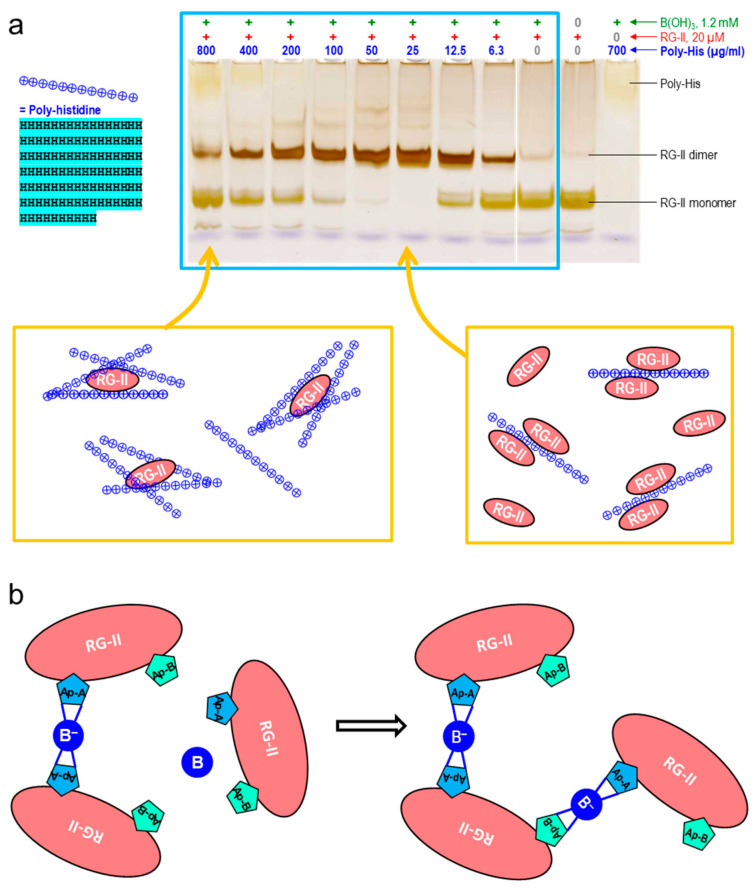
Polyhistidine as a cationic chaperone, catalysing the boron bridging of RG-II. (**a**) Each reaction mixture contained 100 µg/mL RG-II monomer (≈20 µM), 1.2 mM boric acid, 0–800 µg/mL (≈0–44 µM) polyhistidine (chloride salt), and 50 mM acetate (Na^+^) buffer pH 4.8. Controls (outside the blue rectangle) lacked boric acid or RG-II, as indicated above the gels. After 16 h, 0.8 µg of the RG-II was analysed by PAGE followed by silver staining [22]. Below the gel (orange rectangles) is an interpretation of why an optimum polyhistidine concentration exists: at 25 µg/mL, there is an excess of RG-II molecules, which are thus obliged to ‘share’ a polyhistidine molecule bringing them close enough together for dimerisation, whereas at 800 µg/mL, there is an excess of polyhistidine and thus little chance that two RG-II molecules will be brought close together on a single polyhistidine molecule [Gel image adapted with permission from Ref. [22], ©2022, Portland Press]. (**b**) Forming a trimer from a dimer plus a monomer requires at least 1 B bridge to be made through the apiose residue of RG-II’s side chain “B” (Ap-B) rather than through the preferred Ap-A. The trimers and tetramers are unstable: this makes them difficult to isolate, but marks them out as being of biological interest as potentially reversible B bridges. B in a blue circle represents boric acid; B^−^ in a blue circle represents a tetravalent boron atom.

One very effective organic polycation in this context is polyhistidine [55], a synthetic protein composed of only histidine residues. After facilitating the B-bridging of RG-II, polyhistidine was found to depart from the scene, as indicated by the fact that the dimerised RG-II ran on a gel electrophoretogram at the same rate as pure RG-II dimer. In contrast, polylysine remained permanently attached to the RG-II, the complex having a net positive charge such that it migrated towards the cathode and was thus not observed on the gel electrophoretogram [55]. The optimal polyhistidine concentration was low, about 25–50 µg/mL [22] (Figure 6), and polyhistidine-driven dimerisation was maximal at pH 2.5–5.5 [23]. It is likely that at lower pH values, the RG-II loses most of its negative charge whereas at higher pH values the polyhistidine starts to lose its positive charge (Figure 2b): moves in either of these directions would diminish the ionic bonding of anionic RG-II to cationic polyhistidine.

The effect of polyhistidine was dependent on its high M_r_ (a degree of polymerisation, DP, of ~100 was used). Free histidine had no appreciable effect on RG-II dimerisation, even at much higher concentrations (50–1000 µg/mL), indicating that small cations (e.g., histidine itself), like most other free cations tested such as Na^+^, were not able to assist the process of B bridging [22]. An intermediate-sized cation, hexahistidine (His_6_), had some chaperone activity, moderately promoting RG-II dimerisation at 400–1000 mg/mL [22].

Although polyhistidine is not a naturally occurring peptide, Chormova and Fry (2016) also showed that, in addition, a crude preparation of expansins (histidine/lysine-rich cationic glycoproteins of the primary cell wall), was able to promote RG-II dimerization—again acting catalytically as they did not remain attached to the RG-II during electrophoresis [55]. Expansins are tenable candidates to be the natural ‘chaperones’ responsible for B bridging of RG-II in vivo as both these polymer types move through the same endomembrane system and into the cell wall.

**Figure 7:**
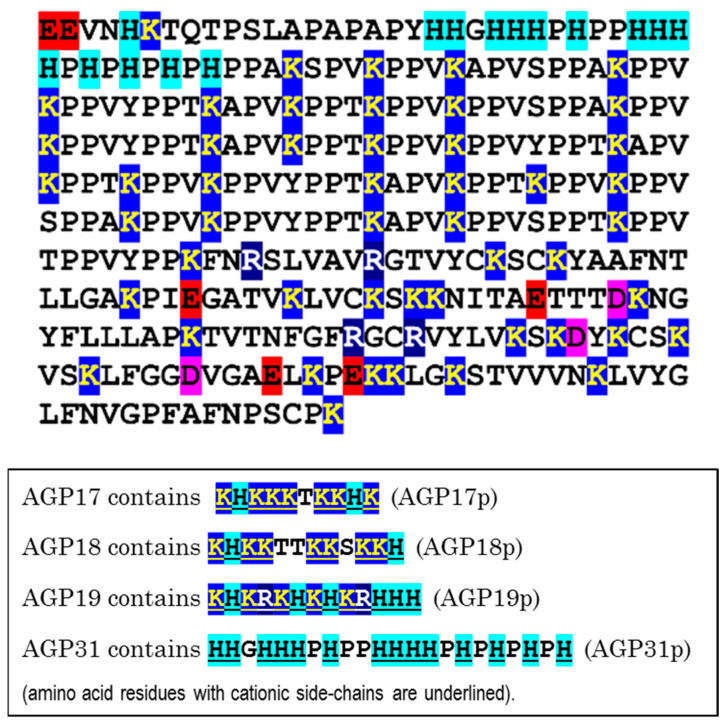
(**Above**) The amino acid sequence of AGP31, with the N-terminal signal peptide removed. The sequence is aligned to emphasise the regular spacing of positively charged lysine (K) and occasional arginine (R) residues. In the native molecule, many of the proline residues (P) are hydroxyproline, and many of those are glycosylated [56,57]. Also shown is the remarkably histidine-rich domain (cyan). Amino acids with charged side-chains are colour-coded as follows: cations in shades of blue (His, cyan; Lys, blue; Arg, dark blue) and anions in shades of red (Asp, pink; Glu, red). The sequence is taken from NCBI database (https://www.ncbi.nlm.nih.gov/protein/OAP15690.1; accessed on 5 September 2022 [58]) [adapted with permission from Ref. [22], ©2022, Portland Press]. (**Below**) Four remarkably basic oligopeptides which appear in arabidopsis arabinogalactan proteins AGP17, 18, 19 and 31.

Besides cationic extensins, the plant cell synthesises and secretes various other glycoproteins that are cationic overall and/or possess a remarkably cationic domain. The arabidopsis genome encodes arabinogalactan proteins (AGPs) that possess such peptide domains (‘AGPps’), shown in the lower half of Figure 7.

Most cationic peptides, e.g., polyhistidine, showed a clear optimum concentration for the B bridging of RG-II (e.g., Figure 6), and it is intriguing to consider why higher concentrations lost effectiveness. One possible explanation might be that each RG-II molecule would bond ionically to excess cations, resulting in complexes with a net positive charge, thus repelling each other electrostatically, just as naked RG-II molecules with a net negative charge repel one another. More likely though, with an excess of the peptide, most peptide molecules would become attached to only one (or zero) RG-II molecules, so the RG-IIs would not be brought close enough together to enable RG-II–B–RG-II bridging. At intermediate polyhistidine concentrations, there was evidently an optimum where the complexes exhibited little electrostatic repulsion and two RG-II molecules would frequently become bonded side-by-side to the same polyhistidine molecule, enabling optimal B bridging (Figure 6a).

This model of RG-II–polycation interaction suggests that two or more RG-II molecules have to simultaneously bond, side-by-side, to a single polycation molecule. Such a scenario, with two RG-IIs seated on a single cationic chaperone molecule, would not be feasible with small chaperones, and indeed small chaperones (e.g., Pb^2+^ and His_6_) did not exhibit a supra-optimal concentration for catalysing B bridging.

Most of the AGP-based cationic peptides tested [AGP17p, AGP18p, AGP19p, (and possibly AGP31p), and the native AGP31 glycoprotein] also exhibited a similar phenomenon to that reported for polyhistidine, each exhibiting optimal concentrations under the experimental conditions used [22]. This is likewise interpreted as evidence for a situation where the RG-II–chaperone complex has little or no net charge (and thus minimal electrostatic repulsion), and two RG-II molecules can simultaneously bind to the same glycoprotein or peptide molecule, as suggested above for polyhistidine.

Thus, certain organic polycations can chaperone RG-II, manoeuvring this polyanionic polysaccharide domain such that B-bridging is favoured. These chaperones dissociate from RG-II after facilitating its dimerization, indicating that they act catalytically rather than stoichiometrically.

Arabidopsis AGP31 is also highly cationic overall (isoelectric point estimated at 9.1) (Figure 7). Curiously, AGP31 has long runs in which there is a (cationic) lysine every 4 or 8 residues, giving the impression of a structure well adapted to ionically bonding to a regular structural polyanion such as a pectic polysaccharide.

It is tempting to suggest that a homogalacturonan–RG-II sequence can lie alongside AGP31 with the homogalacturonan domain ionically bonded along the (KXXX)_n_ stretches and the contiguous RG-II domain being thereby optimally placed to bond ionically to the histidine-rich stretch of the same AGP31 molecule. Indeed, if AGP31 approximates a beta sheet, then its lysine residues along a (KXXX)_n_ stretch come every 1.4 nm, and if it approximates a polyproline-II helix, then they come every 1.24 nm. These values are reasonably close to the pitch (1.34 nm) of a 3_1_ helix of homogalacturonan (=3 GalA residues; [59]). And, given that AGP31 has been reported to self-aggregate in aqueous solution [57], this could enable the AGP31 to chaperone two pectins, bringing two RG-II domains into close proximity.

AGP31 was found to promote RG-II dimerisation in vitro in the presence of B(OH)_3_ [22], leading to the suggestion that this highly cationic glycoprotein can serve as a chaperone, bringing together two RG-II domains ready for B bridging.

The key role of the histidine-rich region of AGP31 in promoting RG-II dimerisation is demonstrated by the observation that this section (HHGHHHPHPPHHHHPHPHPHPH—a 22-amino-acid peptide sequence including an astonishing 14 histidine residues; “Nature’s closest approximation to polyhistidine”?) can by itself serve as a chaperone, promoting the B bridging of purified free RG-II in vitro. It remains to be seen whether the 22-amino-acid peptide can dimerise RG-II domains that are not isolated, i.e., are still glycosidically linked to homogalacturonan as part of a whole pectin macromolecule.

Three other arabidopsis AGPs (AGP17, 18 and 19) also possess histidine-rich regions (sequences given above), and these were also found to promote the B bridging of isolated RG-II domains in vitro [22,23]. Unlike AGP31, the glycoproteins that contain these histidine-rich regions do not possess many other cationic amino acid residues, and therefore the chaperoning function is entirely due to the histidine-rich stretches.

AGP17, 18 and 19 are believed to be held on the outer face of the plasma membrane, and presumably on the inner face of the Golgi cisternae, trans-Golgi network (TGN) and Golgi-derived vesicles prior to secretion, via glycosyl phosphatidyl inositol (GPI) anchors. They are thus in the right place to contribute to RG-II dimerisation before and/or just after secretion, matching the known timing of B bridging [5,23].

Glycoproteins like AGP17, 18, 19 and 31 are not unique to arabidopsis. Similar peptide sequences are also known from numerous diverse land plants [60,61], including lycopodiophytes (https://db.cngb.org/onekp/species/Lycopodium%20deuterodensum; accessed on 22 October 2023). Therefore, the ideas developed here may well be applicable across all land-plants.

### 2.2. The First Known Boron-Acting Enzymes

The effects of AGPs on RG-II dimerisation strongly resemble enzyme action (an RG-II borate diesterase) in many respects. Interestingly, this represents the first report of an enzyme activity capable of acting on B compounds.

Central to its proposed enzyme status, AGP31 is a naturally occurring protein that can catalyse a reaction (Figure 3) resulting in a covalent chemical change between substrates and products.

AGP31 and the histidine-rich oligopeptides are not used up during the reaction, as can be seen in those cases where the chaperone itself is detectable on silver-stained gels (AGP31, AGP31p, polyhistidine and His_6_). The chaperones evidently bind the RG-II substrate reversibly and are released intact after catalysing the reaction. As expected for an enzyme, the chaperones can catalyse the B-bridging of an excess of RG-II: for example, with ~0.5 µM AGP31 acting on 20 µM RG-II. Likewise, modelling this in the case of an artificial polycation, 1.4 mM polyhistidine catalysed the complete dimerisation of 20 µM RG-II—behaviour typical of an enzyme. Furthermore, an oligopeptide fragment of arabidopsis AGP19 catalysed the B bridging of RG-II to close to 100% completion [22].

The reaction under investigation (Figure 3) occurs very slowly in the absence of a catalyst. However, this observation does not detract from the assertion that the cationic glycoproteins and peptides under investigation are enzymic. For comparison, we note that the reactions catalysed by carbonic anhydrase, superoxide dismutase and catalase all readily occur without the help of the respective enzymes:

CO_2_ + H_2_O → HCO_3_^−^ + H^+^

2 H^+^ + 2 O_2_^•−^ → O_2_ + H_2_O_2_

2 H_2_O_2_ → O_2_ + 2 H_2_O

It may be objected that the reaction (Figure 3) can also be catalysed by non-enzymic agents such as lead nitrate; however, this also does not stop AGPs and their oligopeptides being enzymes. For comparison, reactions catalysed by carboxylesterases, given in the general form as ‘ester + water → acid + alcohol’ are mimicked by cold dilute NaOH, and that catalysed by catalase (see above) is mimicked by various inorganic agents such as MnO_2_, PbO_2_ and iron filings. These enzymes do not lose their enzymic status on those grounds.

The reaction (Figure 3) catalysed by AGP31 and cationic oligopeptides is essentially irreversible in the direction ‘acid + alcohol → ester + H_2_O’ (the reverse of the carboxylesterase reaction cited above). These contrasting directionalities are of course not governed by the enzymes but by the thermodynamics of the specific reactions. An enzyme that catalyses this type of reaction in either direction (acid + alcohol ↔ ester + water) is equally an esterase.

In the specific case of interest, the enzyme could be named an RG-II borate diesterase. Equally, it could be called an RG-II borate diester synthase. It is of interest that the Enzyme Commission does not currently list any enzymes acting on B compounds (https://iubmb.qmul.ac.uk/enzyme/EC3/, accessed on 22 October 2023).

With some cationic chaperones (e.g., polyhistidine or Pb^2+^), the dimerisation of RG-II by reaction with boric acid reaches completion; with others (e.g., the highly cationic AGP peptides), it may stop before reaching completion. The failure to reach completion is not due to the reaction reaching an equilibrium at which the rate of dimerisation equals that of re-monomerisation. In fact, a solution of pure dimer did not undergo any detectable monomerisation (even in the presence of chaperones, which might have expedited reaching the equilibrium). Furthermore, the observation that RG-II dimerisation can be catalysed to completion by certain chaperones argues against the hypothesis that an equilibrium is reached. And we assume that the peptides are stable during the (up to) 24-h incubations. Further research will be needed to explain why complete dimerisation is not always reached.

### 2.3. What If Boric Acid Is Not the Boron Donor Substrate?

In studies of the process of B bridging of RG-II, it has routinely been assumed that the B is supplied in the form of simple boric acid. However, the form in which B is available in vivo might be a complex with an organic carrier—potentially a B ‘donor substrate’ for RG-II dimerisation.

Pulse radiolabelling experiments showed that *Rosa* and arabidopsis cell cultures B-bridge their RG-II domains extremely rapidly, presumably in the Golgi (with a small proportion also occurring several hours after deposition within the wall) [23]. Therefore it can be deduced that any natural B-donating agents (e.g., glycoproteins or glycolipids) must be located in the Golgi, TGN, vesicles and plasma membranes.

Chormova and Fry (2016) tested various potential borate-transferring ligands that might help to attach B to RG-II [55]. However, the potential borate-binding ligands examined (apiose, dehydroascorbic acid, alditols) and small organic cations (including polyamines) lacked consistent effects. Possibly other natural water-soluble B donor substrates remain to be discovered.

**Figure 8:**
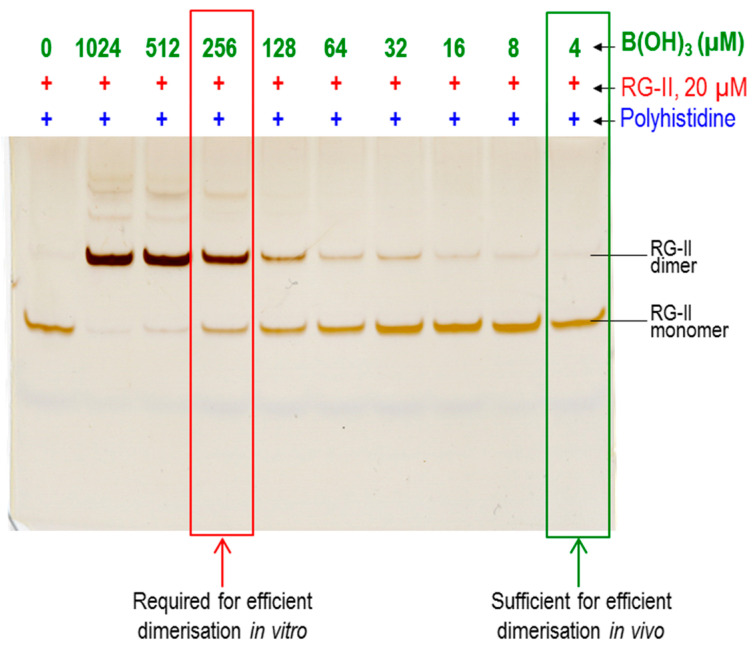
Effect of boron concentration on the polyhistidine-catalysed dimerisation of RG-II [22]. Reaction mixtures initially contained 50 µg/mL monomeric RG-II (20 µM) with 0–1024 µM boric acid and 50 mM acetate (Na^+^, pH 4.8), with polyhistidine (chloride salt; 50 µg/mL; 2.8 µM). After 4 h at 20 °C, the RG-II was analysed by PAGE. Note the difference in B(OH)_3_ concentration required for effective dimerisation in vitro (>250 µM) and that sufficing in the medium of cultured *Rosa* cells (3.3 µM). Adapted with permission from Ref. [22], ©2022, Portland Press.

The concentration of boric acid used to test for RG-II dimerisation in vitro has been rather high: for example, in the presence of polyhistidine (the most effective chaperone found to date), 256 µM B(OH)_3_ was the lowest concentration giving reasonably efficient dimerisation (Figure 8). This can be contrasted with 3.3 µM B(OH)_3_, which is the concentration routinely used in the *Rosa* cell culture medium, in which the cells dimerise most of their RG-II very effectively. It is possible that in vivo the B(OH)_3_ is rendered more effective by means of a B ‘donor substrate’. Alternatively, it is possible that B(OH)_3_ becomes actively concentrated within the Golgi system (taken to include the TGN)—the major location of RG-II dimerisation. It is of interest that the boric acid/borate transporter protein, BOR1, is strongly accumulated in TGN membranes in cells with a low B supply [62].

**Figure 9:**
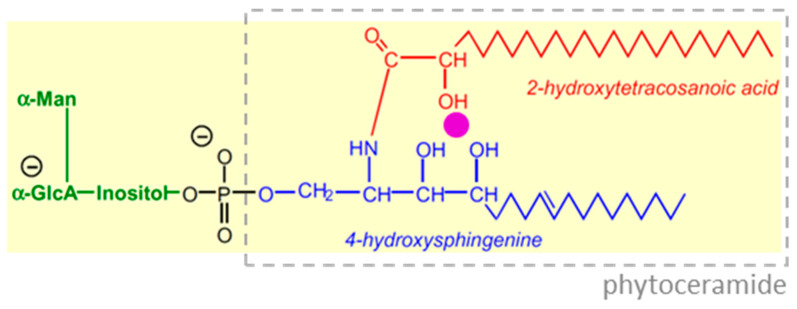
The predominant glycosyl inositol phosphoryl ceramide (GIPC) species found in membranes of *Rosa* cell suspension cultures. Purple dot, potential site of binding of borate to three –OH groups. Green, glycosyl inositol moiety; red, long-chain 2-hydroxy-fatty acid; blue, long-chain base; dashed grey box, phytoceramide moiety. The structure was elucidated by mass spectrometric and thin-layer chromatographic analysis of *Rosa* GIPCs [11]. Abbreviations: Man, d-mannose, GlcA, d-glucuronic acid. Adapted with permission from Ref. [11], ©2014, Wiley Blackwell.

Glycosyl inositol phosphoryl ceramides (GIPCs) are major glycolipids of the plant plasma membrane, associated with lipid rafts [63,64,65]. Their structure, illustrated by the specific case of a GIPC detected in cultured *Rosa* cells (Figure 9; [11]), includes a unique triad of hydroxy groups (two on the C_18_ 4-hydroxysphingenine moiety plus one on the C_24_ fatty acid) which appear well placed to chelate borate (pink circle in Figure 9). The corresponding C_18_ long-chain base in membrane glycolipids of organisms other than plants has only a single unsubstituted hydroxy group (i.e., it is sphingenine rather than 4-hydroxysphingenine) and would therefore be expected to have less affinity for borate.

An ability of plant GIPCs to bind borate is suggested by the observation that in B-starved cells the GIPCs are more easily extracted from the lipid rafts of the membrane [11]. It can therefore be suggested that GIPCs might serve as B donors for RG-II dimerisation, ideally placed in the Golgi- and plasma-membranes. Indeed, the in-vitro formation of a transient GIPC–B–RG-II covalent complex was suggested by the ability of added GIPC + boric acid to slow the mobility of RG-II on high-voltage paper electrophoresis (at pH 2.0 [11]). The complex was broken, returning the [^3^H]RG-II to its original mobility, by cold dilute HCl, which is known to cleave B bridges.

A role for the GIPC–B–RG-II complex in assisting RG-II dimerisation was suggested by a slight enhancement of the dimerisation of RG-II by boric acid in vitro if GIPCs were also added. This observation does not negate the beneficial role of cationic peptides in catalysing RG-II dimerisation. Indeed, a GIPC–B–RG-II complex has a large net negative charge (those routinely present on the RG-II plus one on the esterified borate plus one on the phosphate of the GIPC) which might be expected to repel an incoming second RG-II molecule. Thus, the combination of GIPC + B(OH)_3_ + cationic peptide may act synergistically to augment RG-II dimerisation in vivo. Such multi-component mixtures remain to be tested in vitro.

### 2.4. What If ‘Naked’ RG-II Is Not the Boron Acceptor Substrate?

All in-vitro B-bridging experiments to date appear to have been conducted with purified monomeric RG-II as the substrate. This, however, is not biologically realistic. Free RG-II has not been reported to occur in vivo, although it cannot be ruled out that some free (possibly nascent) RG-II molecules are transiently present within the Golgi system during pectin biosynthesis. As with the above discussion of potential B ‘donor substrates’ it can likewise be concluded that the true B acceptor substrate form of RG-II must be located in the Golgi- and plasma membranes. This is because all in-vivo studies to date ‘view’ EPG-liberated RG-II as the unit investigated, regardless of whether these RG-II molecules were free, homogalacturonan-bound, or RG-I-bound.

In fact, it appears very likely that the substrate for in-vivo B bridging is an RG-II domain that constitutes part of a whole pectin molecule such as

homogalacturonan–RG-II–homogalacturonan

or more complex chains such as

homogalacturonan–RG-II–homogalacturonan–RG-I–homogalacturonan.

Adding to the factors to be taken into account, the homogalacturonan domains in these substrate polysaccharides might be methylesterified (thus uncharged) or de-esterified (thus polyanionic). And in the latter case, the polyanionic homogalacturonans may be calcium bridged, e.g., as ‘egg box’ structures [59,66].

**Figure 10:**
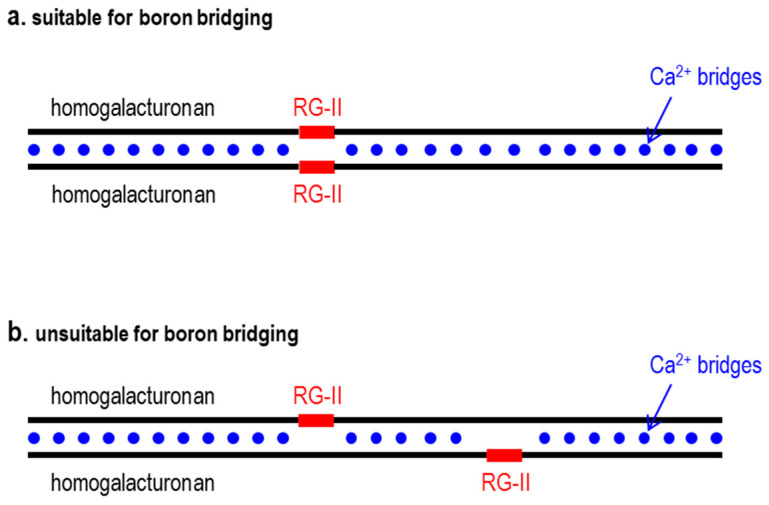
RG-II domains fixed in position by ‘egg-boxed’ homogalacturonan. (**a**) Suitably for B bridging of the RG-IIs. (**b**) unsuitably for B bridging.

All these, and other, factors are likely to influence the intriguing story of the B-bridging of RG-II domains. Calcium bridging of the de-esterified homogalacturonan domains might help a pair of RG-II domains to approach each other close enough for B bridging (Figure 10a). Conversely, such calcium bridging might fix two RG-II domains permanently out of reach of each other (Figure 10b).

Further knowledge of the interactions of all the various players mentioned in this chapter is essential if we are ever to fully understand how plants B-bridge pectin.

## 3. Is RG-II Cross-Linking Reversible In Vivo?

If the B bridging of RG-II plays important roles in dictating the mechanical properties of the cell wall, e.g., its extensibility, then it is of interest to study the occurrence of both RG-II dimerisation and the possible re-monomerisation of previously dimerised RG-II domains.

In fact, pH values at and even below the apoplastic range (2.0–7.0) did not break B-bridges either in vivo or in vitro [23]. When pure free dimeric RG-II was added to zero-B *Rosa* cell cultures in vivo, no loss of the soluble extracellular RG-II dimers was detected over a period of 96 h at 24 °C, and no soluble RG-II monomers appeared [23]. The findings indicate that these B-starved cells would not have had the ability to abstract B from existing apoplastic RG-II dimers. Similar experiments were also conducted in living cultures with the medium buffered at pH 3.5, 4.0, or 4.5. As before, no free RG-II was generated. Thus, extracellular RG-II dimers are stable in vivo, even at physiologically low pH values (characteristic of ‘acid growth’) and under conditions of B starvation.

A wide range of pH values (2–7), for up to 240 h, was studied for their possible ability to cleave RG-II dimers in vitro. Slight loss of both monomeric and dimeric RG-II was noted after 240 h incubation at physiologically extreme acidity (pH 2), although there was no evidence for any dimer → monomer conversion. The partial loss of RG-II was probably due to slight acid hydrolysis of the highly acid-labile apiosyl–GalA linkages of the polysaccharide’s primary structure rather than any cleavage of B bridges, indicating that the B bridges were stable. In conclusion, under the physiological conditions often reported during ‘acid growth’ (e.g., apoplastic pH ~4.5 [67]), dimeric RG-II is neither monomerised nor appreciably hydrolysed.

Thus, B bridges in the plant cell wall are stable under physiologically realistic conditions. It might be argued that plants possibly possess an enzyme capable of cleaving the B bridges of dimeric RG-II. However, other evidence contradicts this: pulse-radiolabelled RG-II domains (endogenously synthesised) quickly dimerised in vivo and the cohort of radiolabelled RG-II domains was never observed to decrease in percent dimerisation. We conclude that B-starved cells cannot salvage traces of B from RG-II, and furthermore that ‘acid growth’ is not achieved by pH-dependent monomerisation of dimeric RG-II.

An interesting twist to this story emerged recently: in the presence of optimal polyhistidine concentrations, RG-II is capable of forming B-bridged trimers and tetramers in addition to the well-known dimers (Figure 2b and Figure 6). The trimers and tetramers were identified by their lower mobility on polyacrylamide gel electrophoresis [23]. Intriguingly, these additional B bridges are very much more labile than those of the dimer. Indeed, it has not so far been possible to isolate the trimers and tetramers intact; when eluted from a polyacrylamide gel and re-electrophoresed, the trimers and tetramers broke down to RG-II dimers.

Forming an RG-II trimer through B bridging of apiose residues must mean that at least one of the two B bridges occurred via a side chain B, which is normally regarded as not contributing to B bridging [28,32,53,68] (Figure 6b). If we represent an RG-II molecule (⬤) as possessing two apiose residues on side chains A and B (thus: Api^A^-⬤-Api^B^), then a classic RG-II dimer can be represented as

Api^B^-❶-Api^A^>**[B**^−^**]**<Api^A^-❷-Api^B^

where ❶ and ❷ are the two participating RG-II monomers, and >**[B**^−^**]**< is the anionic tetrahedral B atom of the borate diester bridge. On this system, the trimer could be, for example

Api^B^-❶-Api^A^>**[B**^−^**]**<Api^A^-❷-Api^B^>**[B**^−^**]**<Api^A^-❸-Api^B^

or similar—the important point being that a (less favoured) side-chain B apiose residue is transiently participating in B bridging. Similar logic can be extended to the observed tetramers.

Therefore, it appears that trimers and tetramers of RG-II, being weak, would contribute reversibly to strengthening the cell wall, and thus it is possible that their cleavage helps to loosen the primary wall and facilitate cell expansion. This represents a novel potential control point in turgor-driven plant growth that cannot be proposed for RG-II dimers. The in-vivo making and breaking of B bridges via the apiose of side chain B is another biologically important area of RG-II research where further investigations will be important.

## 4. Why Is It Important for RG-II to Be Boron-Bridged?

Among all the puzzles about the B bridging of RG-II, the biggest is the enigma of ‘why’ plants do it—what is its selective advantage? It is a process conserved throughout vascular plants, and also occurs to some extent in bryophytes. One way of exploring the benefits conferred by RG-II dimerisation is to withhold B from plants or cell cultures, or to ‘chelate’ out free B(OH)_3_ with azomethine H [17] or 80 mM free apiose [23], and observe their fate.

Curiously, suspension-cultured cells of Paul’s Scarlet rose have been successfully grown since 2009 on a medium containing essentially no B. Whatever roles B-bridged RG-II normally plays in plant cells (indeed, whatever roles B itself plays) can evidently be side-stepped in certain suspension cultures. The Paul’s Scarlet rose culture is a vintage, initiated in 1957 [69] in a relatively normal medium containing ~40 µM total B (taking into account the coconut water added). Later, the culture was taken to Birmingham, where it was kept on ‘MX_1_’ medium containing an unusually low concentration of 3.3 µM B [70]. Since 1975, it has been maintained by Fry in the same 3.3 µM B medium. Possibly this choice fortuitously facilitated the subsequent acclimation of this rose culture in 2009 to a medium with nominally ‘zero’ B (measured value 0.088 µM) [5]. Fleischer et al. (1998) also reported a similar acclimation to ‘zero’ B: after a standard (100 µM B) culture had been taken through 50 sub culturings in 7 µM B, it acquired the subsequent ability to grow indefinitely in the absence of detectable B (<0.1 µM B) [6]. We are not aware of any reports of whole plants growing in the complete absence of B, so possibly the B bridging of RG-II is a process essential for whole plants that can be circumvented in cell cultures. A precedent for this type of phenomenon is in the ability to obtain certain cell suspension cultures lacking cellulose [71,72,73] if acclimated to grow in the presence of 2,6-dichlorobenzonitrile, a cellulose synthesis inhibitor, whereas no whole plants have been grown to maturity without cellulose. In the study by Shedletzky et al. (1992), the culture’s cellulose appeared to have been replaced by high pectin plus phenolics, which perhaps conferred the mechanical strength normally endowed by cellulose [71]. It remains to be seen whether zero-B rose cell cultures exhibit some cell wall modification compensating for the lack of dimeric RG-II.

Another way of exploring the benefits conferred by RG-II dimerisation is to study mutants with slightly modified RG-II primary structures, which have a decreased ability to bind B, especially when the plants are given a relatively low-B medium. For example, the tobacco mutant *nolac-H18* (defective in NpGUT1, a glucuronosyltransferase), which lacks GlcA and l-Gal in sidechain A, has diminished RG-II B bridging [74]. The arabidopsis mutants *mur-1* and *sfr8* (replacing l-Fuc with l-Gal in sidechain A) are defective in B-mediated RG-II dimerisation owing to the truncated sidechain A [19,43,75]. Both *bor1* [76] and *mur1* mutants show dwarfism when B deficient. An arabidopsis mutant unable to produce KDO, a unique monosaccharide of RG-II, was shown to be defective in pollen tube formation and elongation [77]. The *NbAXS1* mutant (virus-induced silencing of a UDP-d-apiose/UDP-d-xylose synthase gene in *Nicotiana benthamiana*), which is unable to synthesise enough d-apiose, 2-*O*-methyl-l-fucose and 2-*O*-methyl-d-xylose, suffers cell death [78]. A recent study showed that UDP-d-apiose/UDP-d-xylose synthase (AXS) in *Arabidopsis thaliana* is encoded by two homologous genes, *AXS1* and *AXS2*, and the *axs* double mutation is lethal, whereas the heterozygotes develop different symptoms; for example, *axs1*/+ *axs2* plants are unable to produce seeds and eventually die in contrast to the *axs1 axs2*/+ plants, which show loss of shoot and root apical dominance [79].

Whichever means are used to block RG-II dimerisation, the outcomes are essentially the same: dwarfism, restricted cell expansion, defective wall permeability, and decreased freezing tolerance. The diminished cell expansion due to a defect in B cross-linking of RG-II in pumpkin reported by Ishii et al. (2001) is curious because a cross-linking process in the primary cell wall might be expected to increase the wall’s coherence and thus to decrease its extensibility at any given turgor pressure [80]. What we have here, though, is a wall cross-linking process that seems to favour the ability of the wall to stretch irreversibly (i.e., of the cell to grow).

Currently, we have no explanation for this apparent paradox, and an important future challenge will be to solve it.

We would, however, note that the low concentration of RG-II in the cell wall does not negate it as a candidate to dictate the wall’s biophysical properties. If pectin really is a long polysaccharide molecule of the type ‘……HG–RG-I–HG–RG-II–HG–RG-I–HG……’, then even a rare inter-molecular cross-link (RG-II–B–RG-II) could significantly tighten the cell wall through the (essentially untearable!) covalent borate diester bond.

## 5. Conclusions and Future Directions

This review summarises current knowledge and ideas on RG-II and its B-mediated dimerisation. There are still many unanswered questions to be explored to reveal the biosynthesis, incorporation into the complex multi-domain polysaccharide known as ‘pectin’, post-synthetic modifications, and—especially—the exact biological functions of RG-II. The only fully defined chemical reaction of B with organic matter in plants is to covalently dimerise RG-II. However, why dysfunctions in this process cause plants to suffer dwarfism, infertility and even death remains mysterious. Nevertheless, the available data emphasise the importance of B-mediated cross-linking of RG-II for the survival of plants.

Our own main contribution to the B–RG-II story has focused on the proposal that RG-II biosynthesis in the Golgi bodies is very close—spatially and temporally—to B bridging. Studying positively charged AGP fragments has revealed these as plausible biocatalysts necessary for RG-II dimerisation. Understanding the underlying mechanisms of RG-II biosynthesis and B-bridging will help us to understand the evolution and mystery of the presence of this conserved complex carbohydrate in the plant cell wall.

## Data Availability

All novel data are included in this manuscript; all other data are from the references cited.
